# Boosting Numerical Cognition in Children and Adolescents with Mathematical Learning Disabilities by a Brain-Based Intervention: A Study Protocol for a Randomized, Sham-Controlled Clinical Trial

**DOI:** 10.3390/ijerph182010969

**Published:** 2021-10-19

**Authors:** Giulia Lazzaro, Andrea Battisti, Cristiana Varuzza, Laura Celestini, Pierpaolo Pani, Floriana Costanzo, Stefano Vicari, Roi Cohen Kadosh, Deny Menghini

**Affiliations:** 1 Child and Adolescent Neuropsychiatry Unit, Department of Neuroscience, Bambino Gesù Children’s Hospital, IRCCS, 00146 Rome, Italy; giulia.lazzaro@opbg.net (G.L.); andrea.battistidys@gmail.com (A.B.); cristiana.varuzza@opbg.net (C.V.); lau.celestini@gmail.com (L.C.); floriana.costanzo@opbg.net (F.C.); stefano.vicari@opbg.net (S.V.); 2 Department of Human Science, LUMSA University, 00193 Rome, Italy; 3 Department of Physiology and Pharmacology, Sapienza University, 00185 Rome, Italy; pierpaolo.pani@uniroma1.it; 4 Department of Life Sciences and Public Health, Università Cattolica del Sacro Cuore, 00168 Rome, Italy; 5 School of Psychology, Faculty of Health and Medical Sciences, 30AD04 Elizabeth Fry Building, University of Surrey, Guildford GU2 7XH, UK; r.cohenkadosh@surrey.ac.uk; 6 Department of Experimental Psychology, University of Oxford, New Radcliffe House, Radcliffe Observatory Quarter, Oxford OX2 6GG, UK

**Keywords:** specific learning disorders, transcranial electrical stimulation, EEG, evidence-based medicine

## Abstract

Numbers are everywhere, and supporting difficulties in numerical cognition (e.g., mathematical learning disability (MLD)) in a timely, effective manner is critical for their daily use. To date, only low-efficacy cognitive-based interventions are available. The extensive data on the neurobiology of MLD have increased interest in brain-directed approaches. The overarching goal of this study protocol is to provide the scientific foundation for devising brain-based and evidence-based treatments in children and adolescents with MLD. In this double-blind, between-subject, sham-controlled, randomized clinical trial, transcranial random noise stimulation (tRNS) plus cognitive training will be delivered to participants. Arithmetic, neuropsychological, psychological, and electrophysiological measures will be collected at baseline (T0), at the end of the interventions (T1), one week (T2) and three months later (T3). We expect that tRNS plus cognitive training will significantly improve arithmetic measures at T1 and at each follow-up (T2, T3) compared with placebo and that such improvements will correlate robustly and positively with changes in the neuropsychological, psychological, and electrophysiological measures. We firmly believe that this clinical trial will produce reliable and positive results to accelerate the validation of brain-based treatments for MLD that have the potential to impact quality of life.

## 1. Introduction

Mathematical learning disability (MLD), well known as dyscalculia, is a neurodevelopmental disorder that impairs an individual’s ability to learn number-related concepts, perform accurate math calculations, and solve math problems [1]. With a current prevalence of ~7% worldwide [2], MLD persists into adulthood and represents a growing phenomenon. In Italy, its diagnosis increased 160% from 2017 to 2019 [3].

Low numerical competence can prevent high quality-of-life standards throughout development [4,5,6,7], with potential long-term consequences in the professional, psychological, and social arenas. Low mathematical abilities correlate highly with poorer academic and working success [8,9], restricted use of health resources [10,11,12], worse socioeconomic status, and individuals with MLD usually experience considerable amounts of stress and negative feelings in educational settings. When MLD is not recognized and treated, negative school experiences and a repeated lack of success in mathematical tasks can generate fears of failure and lower self-esteem [13]. Moreover, the impact of MLD in the onset of later psychopathological symptoms (e.g., anxiety disorder, depression) is well described [13]. MLD also requires extreme annual expenses to counteract its harmful societal consequences [14].

Nevertheless, current therapeutic options do not provide sufficient support to children with MLD and their families [13,15].

### 1.1. Neurocognitive Features of MLD

When a child is asked to solve an apparently simple arithmetic operation, multiple neurocognitive processes are rapidly encompassed. One way to improve mathematical abilities is by understanding such underlying neurocognitive functions.

On the cognitive level, experimental evidence suggests that “core” and “noncore” skills are impaired in individuals with MLD [16,17]. Core skills refer to the innate ability to process numerical information without consciously dealing with symbolic representations of numbers and are shared between humans (including infants and indigenous tribes who have little or no formal mathematical education [18,19]) and other species (for a review, see [20,21,22]). Core skills are considered to be specialized for such mathematical abilities as automaticity in processing numerical information, the ability to discriminate numerosities, representation of numerosities, mapping numbers onto space, and counting [23]. This sense of numerosity (also known as number sense [24]) has been widely proposed as a foundational basis for higher-order cognitive processes in the acquisition of abstract numerical concepts and in later achievements in mathematics [25,26,27,28].

In contrast, noncore skills are those that are crucial for numerical cognition but are not exclusive to the mathematical domain, such as executive functions (e.g., inhibitory control, working memory), visuospatial skills (e.g., mental rotation, visuospatial reasoning), and attention [29,30,31,32,33,34]. Theoretical evidence shows that working memory and visuospatial reasoning strongly predict mathematical achievements [35,36,37]. Working memory (likely the most widely studied domain-general function in relation to mathematical abilities [for a review, see 35]) has been linked to an individual’s ability with regard to fact retrieval, manipulation of operators, operands, and numerical facts [38,39]. Notably, a large community-based study by Judd and Klingberg [34] demonstrated that visuospatial training can be transferred to academic abilities and that reasoning ability and maintenance of spatial information are relevant for learning mathematics in young children.

On the neural level, an abnormal bilateral frontoparietal network has been consistently reported in individuals with MLD [40]. Specifically, atypical brain function has been found in the posterior parietal cortex (PPC) during numerical processing and calculation tasks [41,42] and in the temporoparietal areas during number facts, which requires the automatic retrieval of verbally stored numerical information from long-term memory [43]. Further, abnormal activation has been observed in the prefrontal regions, including the dorsolateral prefrontal cortex (dlPFC), regions that orchestrate noncore domain-general processes (such as working memory and problem solving) [44,45,46].

### 1.2. Current and New Treatment Perspectives in MLD

Given the profound negative effects of MLD, significant efforts have been made to develop effective interventions. In the last 20 years, educational programs [47,48,49], tutoring [50], and cognitive-based training programs [13] have been proposed to support children and adolescents with MLD. Although certain cognitive-based interventions have affected improvements [51,52,53], the results remain inconsistent and have not been examined systematically and integrated into a unique therapeutic practice [13]. Their efficacy and long-term outcomes remain unknown [52], and most studies are not supported by empirical and peer-reviewed research [54]. Collectively, the results for cognitive-based interventions lack high-quality standardized and evidence-based methods for clinical translation in interventional settings [13].

Extensive data from recent years on the neurobiology of MLD have increased the interest in neurostimulation approaches (e.g., transcranial random noise stimulation-tRNS), based on their potential to manipulate brain networks directly, alone or by enhancing the effects of other interventions [55]. tRNS is a safe, painless, cost-effective, affordable, portable, and user-friendly treatment option for the pediatric population. It is a polarity-independent form of transcranial electrical stimulation that entails the application of a weak current to the scalp at random intensities (e.g., ±0.5 mA) over a wide range of frequencies (from 0.1 to 640 Hz) [56].

A phenomenon, called stochastic resonance, would explain the mechanism of tRNS [57] and refers to the amplifying effect of adding noise to a signal that is too weak to exceed a threshold on its own [58]. However, its mechanism at the neural level remains under debate [59]. tRNS likely boosts long-term potentiation-like cortical plasticity by inducing the repetitive opening of sodium channels, shortening the hyperpolarization phase [58,59,60]. Moreover, a recent study in juvenile mice [61] has suggested that the effects of tRNS are attributed to modulation of the precursor of GABA, a neurotransmitter that is involved in neuroplasticity. tRNS could enhance excitability, which underlies the atypical bilateral frontoparietal network in children with MLD, with the potential to desynchronize dysfunctional rhythms.

Although the literature is increasingly highlighting the successful application of tRNS in enhancing arithmetic learning in healthy adults [62,63,64,65], our understanding of its real-world translation to clinical settings (particularly in atypically developing children) remains poor. Only a single-blind, between-subject pilot study has examined the effects of 4 sessions of tRNS over 10 days of cognitive training compared with placebo in children with MLD [66]. Twelve participants were pseudorandomized to receive active or sham tRNS over their bilateral dlPFCs while they performed a concomitant number line training. Active tRNS was beneficial compared with sham tRNS in improving arithmetic learning and performance while being safe and tolerable in the pediatric population [66].

Given the preliminary nature of the aforementioned study [66], open questions remain regarding the most suitable, effective, and feasible tRNS protocol for improving arithmetic abilities in a wide sample of children and adolescents with MLD (for example, the appropriate number of sessions, the most effective placement of electrodes (e.g., PPC vs. dlPFC), and the electrophysiological effects of tRNS).

Based on these encouraging preliminary results [66], large-scale and high-reproducibility clinical trials are urgently needed. According to the National Institute of Mental Health, insufficient reporting of study protocols is a critical aspect that hinders the development of therapeutic applications in neurostimulation [67].

### 1.3. Research Objectives

The overarching goal is to provide a scientific foundation for devising brain-directed and evidence-based treatments in children and adolescents with MLD. Based on insights from recent empirical research, this study addresses at least five gaps in research:

(1) Examining the long-term safety and feasibility of a multisession tRNS protocol in the pediatric population (e.g., 10 sessions);
(2) Determining the effects of the tRNS setup (over bilateral dlPFCs vs. bilateral PPCs) in improving long-lasting arithmetic learning and performance and neuropsychological and psychological measures compared with sham tRNS;
(3) Testing whether and the extent to which arithmetic improvements after tRNS are related to neuropsychological changes;
(4) Understanding whether and the extent to which changes in spontaneous EEG after tRNS are linked to arithmetic improvements;
(5) Assessing whether and the extent to which improvements in arithmetic performance correlate with changes in the psychological function of children and their parents’ stress.

## 2. Materials and Methods

### 2.1. Ethical Committee

Ethical approval for this study was granted by the local research ethics committee (process number 1547_OPBG_2018) and was registered at ClinicalTrials.gov (ID: NCT04242680) on 28 January 2020. This study will be performed in accordance with the Declaration of Helsinki. The protocol adheres to the SPIRIT guidelines (Standard Protocol Items: Recommendations for Interventional Trials) and was prepared using the SPIRIT 2013 Checklist [68].

### 2.2. Study Setting and Participants

Recruitment will take place at the Child and Adolescent Neuropsychiatry Unit of Bambino Gesù Children’s Hospital in Rome. Participants will be enrolled during the daily clinical activities of the I.Re.Ne Lab (Innovation Rehabilitation in Neurodevelopment Lab) by psychologists, neuropsychiatrists, and speech therapists. Should that be the case, participants will be selected retrospectively from a comprehensive database, upheld by the direction of the Head of the Unit (S.V.), which encompasses several hundred patients who have been assessed according to the good clinical practices per international guidelines for neurodevelopmental disorders. Research assistants will contact the selected participants by phone and e-mail to determine their interest in the project.

All participants and their parents will be fully informed of the procedures and purpose of the experiment, and the principal investigator will obtain written consent prior to participant entrance into the study. Participation will be solely voluntary.

The inclusion criteria are as follows: (1) participants of both genders, diagnosed with MLD per the Diagnostic and Statistical Manual of Mental Disorders, Fifth Edition (DSM-5) [1] and national recommendations [69], confirmed by experienced developmental psychiatrists and psychologists through their developmental history and extensive clinical examination; (2) the mathematical total quotient (MTq), per the Diagnostic Battery for Developmental Dyscalculia, Second Edition (BDE-2) [70], at least two standard deviations (SDs) below the mean (composite score ≤ 70); (3) intelligence quotient (IQ) ≥ 85; (4) age between 8 years and 6 months and 14 years and 11 months inclusive; and (5) normal or corrected-to-normal vision.

The exclusion criteria are: (1) the presence of another primary psychiatric diagnosis (e.g., depression, anxiety), autism, or attention deficit hyperactivity disorder; (2) a personal history of neurological/medical/genetic diseases; (3) a personal history or first-degree-relative history of epilepsy; (4) having undergone neuropsychological treatment for MLD in the year before the baseline screen; and (5) receipt of a CNS-active drug treatment.

### 2.3. Design, Randomization, and Blinding

The study will use a between-subject, randomized, double-blind, and sham-controlled design.

The screen for clinical eligibility will be completed on Day 0 of the trial, before the stratified randomization. Randomization occurs immediately after a participant completes the screening assessment, ensuring concealment of allocation. Participants will be allocated randomly to the following conditions: (1) Frontal Group (active tRNS over bilateral dlPFCs + cognitive training); (2) Parietal Group (active tRNS over bilateral PPCs + cognitive training); and (3) Sham Group (Sham tRNS over bilateral dlPFCs/PPCs + cognitive training). An independent researcher will perform the stratified randomization. The stratified randomization will use the minimal sufficient balancing method to prevent imbalances in baseline and will be based on the participants’ demographics (e.g., age, IQ, gender) and MTq [70].

The investigators who assess the participants will be blinded to the allocation, as will the participants and their parents. An independent researcher will maintain the randomization information until the data collection is completed. An emergency code break envelope will be provided to the principal investigator and will only be opened in the case of an emergency, such as a serious adverse event that requires knowledge of the interventions to manage the participant’s condition.

**Figure 1:**
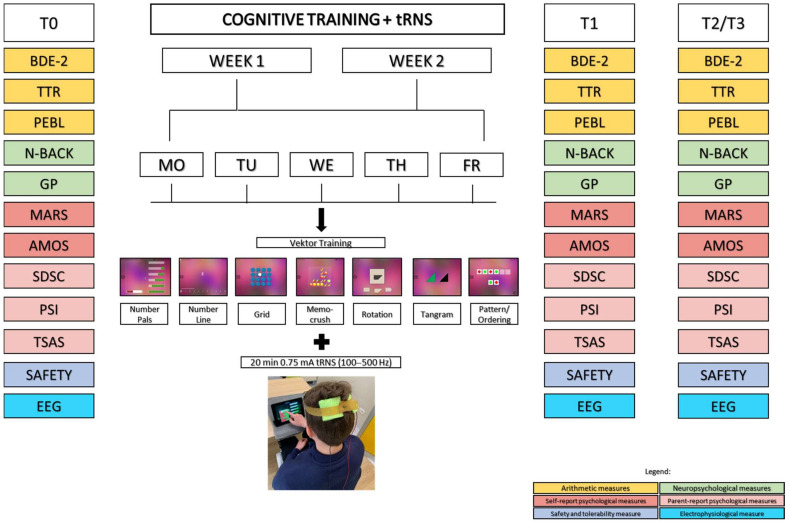
Overview of the study design. T0, Baseline; T1, immediately after the end of the treatment; T2, 1 week after the end of the treatment; T3, 3 months after the end of the treatment; tRNS, transcranial random noise stimulation; MO, Monday; TU, Tuesday; WE, Wednesday; TH, Thursday; FR, Friday; BDE-2, Diagnostic Battery for Developmental Dyscalculia, Second Edition; TTR, Tempo Test Rekenen; PEBL, Psychology Experiment Building Language software; N-BACK; GP, Geometric Puzzle (subtest of the NEPSY); MARS, Mathematics Anxiety Rating Scale-Revised; AMOS, ability and motivation to study; SDSC, Sleep Disturbance Scale for Children; PSI, Parent Stress Index; TSAS, Transverse Symptoms Assessment Scale (questionnaire from the Kiddie-SADS present and lifetime versions, Diagnostic and Statistical Manual of Mental Disorders 5); safety and tolerability questionnaire; EEG, electroencephalogram. Images adapted from Cognition Matters.

A baseline assessment (see Figure 1) will be completed on Day 1 (T0), before the interventions are administered. Participants will undergo combined interventions for 10 days (5 consecutive days per week for 2 consecutive weeks: from Day 1 to Day 5 and from Day 8 to Day 12). To minimize the influence of intracircadian variations, participants will attend stimulation sessions each day at the same time. Participants will also complete an assessment at the end of the interventions (Day 12, T1), 1 week later (Day 19, T2), and 3 months later (Day 102, T3). At the last follow-up, after the assessment, participants will be asked to guess their stimulation condition by the principal investigator.

### 2.4. Interventions

#### 2.4.1. Cognitive Training

Vektor Cognition Matters (https://cognitionmatters.org/, accessed on 5 October 2021) [34,71] is a challenging open-source and age-based training software program that is designed to support and improve the learning of math in children and adolescents. Vektor consists of several domain-specific (numerical abilities) and domain-general tasks (working memory, mental rotation, and logical reasoning) that are automatically and equally alternated for 20 min. As an adaptive training program, it is calibrated to each individual’s performance.

#### 2.4.2. Transcranial Random Noise Stimulation

**Figure 2:**
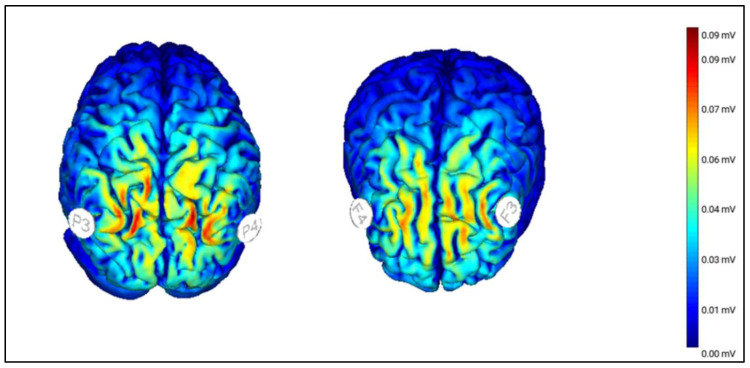
Map of electric field magnitudes in a male brain model from the frontal (**right**) and parietal views (**left**). Stimulating electrodes will be placed over the bilateral dlPFCs (**right**) and bilateral PPCs (**left**) and will be induced with a current with amplitudes varying randomly between −0.375 and 0.375 mA while oscillating at frequencies between 100 and 500 Hz. The actual stimulation will last for 20 min, whereas the sham stimulation will consist of a current ramping up and down within 30 s.

Participants in the active tRNS groups will receive 0.75 mA (±0.375 mA) of tRNS (100–500 Hz) to their bilateral dlPFCs (Frontal Group) or bilateral PPCs (Parietal Group) via 2 saline-soaked 25-cm^2^ sponges, placed over F3/F4 or P3/P4, respectively, per the International 10–20 System (Figure 2). The current will be delivered by a BrainStim stimulator (E.M.S. s.r.l.; Bologna, Italy) for 20 min per session, as in previous tRNS protocols [62,63,64,65,66,72,73] and in transcranial direct current stimulation (tDCS) [74,75,76,77] in pediatrics. As in earlier tDCS studies [74,75,76,77], a low dose (1/8 of an inch) of gel cream will be applied to the sponge’s surface to reduce the likelihood of irritation due to electrical stimulation. The impedance of the electrodes will be checked before and during the application of tRNS to ensure that it remains below 10 kΩ.

To control for any placebo effects, participants in the Sham Group will undergo the same procedures as those in the active groups (Frontal Group, Parietal Group), including the same electrode placement, actual placement of electrodes, and time for powering up the tRNS equipment (30 s). Other than this brief stimulation, participants who are randomized to sham tRNS will not receive active stimulation (0 mA) during the remainder of the session. All children will be blinded to the stimulation conditions. See Figure 2.

### 2.5. Outcome Measures

As described, the outcome measures will be presented to each participant individually at T0, T1, and each follow-up (T2 and T3) in a quiet room in random order to control for the effects of fatigue. The outcome measures will include arithmetic, neuropsychological, self-administered, and parental psychological questionnaires and electrophysiological measures.

#### 2.5.1. Arithmetic Measures

Battery for Developmental Dyscalculia. The primary outcome of the study will be the Number Line subtest of BDE-2 [70]. BDE-2 [70] is a standardized clinical battery that is usually adopted in Italy for evaluating numerical cognition in children between the third grade of primary school to the third grade of secondary school. The battery encompasses several subtests that, in turn, are divided into three main quotients: number, calculation, and number sense and a comprehensive index of math abilities, the MTq. Higher scores indicate better outcomes and will be considered as an improvement.

Tempo Test Rekenen (TTR). TTR [78] measures the ability of children to correctly answer with no overt signs of calculations but automatically retrieving arithmetic information from long-term memory. The test consists of 200 written arithmetic number facts, divided into 5 subtests: addition (e.g., “2 + 3 = _”), subtraction (e.g., “8 − 3 = _”), multiplication (e.g., “5 × 9 =_”), division (e.g., “15 : 3 =_”), and mixed facts (e.g., “2 + 1 =_”; “2 − 1 =_”; “2 × 5 =_”; “4 : 2 =_”). Each subtest contains eight blocks of five operations that are presented with increasing difficulty, and participants are asked to solve as many arithmetic facts as possible in a maximum of 60 s for each subtest. The total number of correct answers is considered. Higher scores reflect better outcomes and will be considered as an improvement.

Math Processing Task. This task is selected from the Psychology Experiment Building Language program (http://pebl.sourceforge.net/, accessed on 5 October 2021) [79,80,81], which measures the ability of children to approximately quantify the numerical magnitude of arithmetic information that is automatically retrieved from the long-term memory. Arithmetic facts (addition and subtraction) with single-digit numbers are presented at the center of a PC screen in horizontal format as a solution from left to right, followed by an equal sign (e.g., “8 + 1 =_”; “9 − 3 =_”). Participants are required to determine whether the result is less or greater than five by pressing the left shift key (for results less than five) or the right key on the keyboard (for results more than five). Several arithmetic facts are presented in 60 s, and participants are required to respond within 1500 milliseconds (ms).

At the end, the following data are automatically recorded: (a) accuracy of each response (0 = incorrect within 1500 ms or correct but after 1500 ms; 1 = correct within 1500 ms) and (b) reaction times (in ms) for each response. With regard to accuracy, higher scores indicate better outcomes and will be considered as an improvement. For speed, lower scores reflect better outcomes and will be considered as an improvement.

#### 2.5.2. Neuropsychological Measures

N-back [82] is a working memory task that comprises two conditions: visual-spatial and verbal. The visual-spatial condition consists of a series of visual stimuli (black boxes) in a certain location on the screen. After a training phase, participants are required to indicate whether the location of each box is the same as that of the box that immediately preceded it (level: 1-back). When the accuracy becomes greater than or equal to 80%, the evaluator will increase the difficulty of the n-back (for example, advancing from 1-back to 2-back). Similarly, the verbal condition entails listening to a continuous stream of letters. After a training phase, participants are required to decide whether each letter matches the letter that was heard immediately before (level: 1-back). When the accuracy becomes greater than or equal to 80%, the evaluator will increase the difficulty of the n-back (for example, progressing from 1-back to 2-back).

For both conditions, 35 trials with an interval between stimuli of 3.20 s will be presented. The number of correct answers and errors is considered and will be used to calculate the accuracy percentage on each level of n-back. Higher scores indicate better outcomes and will be considered as an improvement.

Geometric Puzzle. This subtest, as part of the NEPSY, Second Edition [83], measures mental rotation abilities in children and adolescents aged 3 to 16 years. A grid that contains several blank geometric figures in the center of the page is shown with a number of black geometric figures at the bottom. Participants are asked to pair two of the black figures to the two blank figures inside of the grid in a maximum of 45 s. First, participants are given instructions for the test and two practice items. Mental rotation abilities are necessary when the same figures inside and outside of the square are not in the same position, one of which has been rotated. The number of correct answers is recorded, and the raw score is converted into a weighted score. Higher scores reflect better outcomes and will be considered as an improvement.

#### 2.5.3. Self-Administered Psychological Questionnaires

Mathematics Anxiety Rating Scale-Revised (MARS-R), Italian adaptation. The Italian adaptation of the MARS-R [84] is a self-administrated questionnaire that is composed of 30 items that measures 3 main areas: learning math anxiety, math evaluation anxiety, and school anxiety. Participants are asked to read each item (e.g., “observe a professor working on the blackboard on an algebraic equation”) and indicate his level of anxiety on a Likert scale from 1 (low) to 4 (high). The raw scores are converted into z-scores. Lower scores indicate better outcomes and will be considered as an improvement.

Ability and motivation to study (Test AMOS 8-15). This battery measures strategies and motivation to learn in students aged 8 to 15 years to identify dysfunctional attitudes. It is based on a multicomponent metacognitive model [85] and considers a complex set of relationships that exist between metacognitive, strategic, cognitive, and emotional-motivational factors that collectively determine the level of academic achievement. The battery is composed of five questionnaires as follows: (1) Study Approach Questionnaire (QAS); (2) Questionnaires on Utility and Use of Strategies study (QS1 and QS2); (3) Study tests (PS); (4) Belief Questionnaires (QC1I, QC2F, QC30); and (5) Attributions (QCA). Each questionnaire can be administered alone or combined with others. In this study, only QAS, QC1I, and QC2F will be considered.

QAS is composed of 49 items, 7 each for 7 areas: motivation, organization, personal development, study flexibility, concentration, school anxiety, and attitude toward school. Participants are asked to read each item (e.g., “I like studying to learn new things”) and express their level of agreement on a Likert scale from 1 (not very true) to 3 (very true). QC1I comprises 4 items and evaluates personal beliefs on intelligence (static intelligence vs. modifiable intelligence). Participants are asked to read each item (e.g., “Your intelligence is something about you that you can not change”) and express their level of agreement on a Likert scale from 1 (not very true) to 3 (very true). QC2F is composed of three items and assesses personal beliefs with regard to their intellectual level or abilities/personality. Participants are asked to read each item (e.g., “I usually think I’m smart”) and express their level of agreement on a Likert scale from 1 (not very true) to 3 (very true). 

For all questionnaires, raw scores are converted into z-scores. Higher scores indicate better outcomes and will be considered as an improvement.

#### 2.5.4. Parental Psychological Questionnaires

Sleep Disturbance Scale for Children (SDSC). The SDSC questionnaire [86] evaluates specific sleep disorders and provides an overall measure of sleep disturbances, suitable for use in clinical screens and research in populations aged 6 to 15 years. It consists of 26 items and examines 6 main categories that represent some of the most common sleep difficulties that affect children and adolescents: disorders of initiating and maintaining sleep, sleep breathing disorders, disorders of arousal/nightmares, sleep-wake transition disorders, disorders of excessive somnolence, and sleep hyperhidrosis (night-time sweating).

Parents are asked to read each item (e.g., “the child has difficulty falling asleep”) and indicate how frequently certain behaviors are exhibited by their children on a Likert scale from 1 (“never”) to 5 (“always”). Parents are also required to provide estimates of sleep quantity and time of onset in their children. For all categories, the raw scores are converted into t-scores. Lower scores reflect better outcomes and will be considered as an improvement.

Parent Stress Index (PSI), Italian adaptation. This questionnaire is the most widely used survey for assessing parental stress in clinical and research settings. The Italian adaptation of the PSI [87] is composed of 36 items and examines 3 main areas: parental distress, parent-child dysfunctional interaction, and difficult child. Parents are asked to read each item (e.g., “I often have the feeling of not being able to cope very well with situations”) and indicate their level of agreement on a Likert scale from 1 (“strong agreement”) to 5 (“strong disagreement”). For all areas, the raw scores are converted into z-scores. Higher scores signify better outcomes and will be considered as an improvement.

Transverse Symptoms Assessment Scale. This questionnaire is part of the Kiddie-SADS present and lifetime versions per the Diagnostic and Statistical Manual of Mental Disorders 5 [88]. It comprises 25 items and evaluates health and the relevant symptoms that are related to psychiatric disorders (depression, anger, irritability, mania, anxiety, somatic symptoms, carelessness, suicidal ideation/suicide attempt, psychosis, alterations in sleep, repetitive thoughts and behaviors, and substance use), yielding a comprehensive clinical picture of youth aged 6 to 17 years.

Parents are asked to read each item (e.g., “Did he/she look angry or lose his temper?”) and indicate how much or how often the child has exhibited specific symptoms in the last two weeks on a five-point Likert scale from 0 (“Absent or not at all”) to 4 (“Severe or almost every day”). Items that are related to suicidal ideation, suicide attempts, and use of substances are answered “Yes”, “No”, or “I don’t know”. Consistently high scores in a particular domain might indicate significant and problematic symptoms for the participant, which could justify further evaluation, treatment, and follow-up.

#### 2.5.5. Electrophysiological Measures (EEG)

EEG data will be collected at T0 (immediately before the first session of the interventions), T1 (immediately after the last session), T2, and T3 (before administration of the arithmetic, neuropsychological, and psychological measures). The data will be collected via Geltrode electrodes using a Starstim device (8 channels: AF7, AF8, F3, F4, P3, P4, P7, P8 sites, Neuroelectrics) at a sampling rate of 500 Hz for 5 min in the resting state with eyes closed.

The Geltrode is a wet EEG electrode that provides a rear-fill aperture for the gel supply. It requires a conductive electrode gel and can be used in scalp areas with or without hair. The Geltrode has an Ag/AgCl-coated core that is 12 mm in diameter. It has a rear-fill aperture, and the contact area is approximately 1 cm^2^. The Geltrode electrode in the desired position of the wireless cap will be inserted. After the cap is placed on the head of the participant, a curved syringe will be used to inject the conductive gel through the hole on top of the electrode. Impedance will be kept below 10 kΩ. We will use EEG data in an exploratory manner to detect differences in spectral power frequencies. The EEG will serve as a noninvasive, objective biomarker for tRNS-induced effects and will be considered to be a correlate of behavioral improvements.

#### 2.5.6. Safety and Tolerability

Symptoms and side effects will be assessed using a standard questionnaire [89] to be completed by participants after each session and at each follow-up (T1, T2, and T3). The questionnaire lists adverse effects, such as headache, neck pain, scalp pain, tingling, itching, burning sensation, skin redness, sleepiness, trouble concentrating, and acute mood change. Participants will quantify the intensity of the symptoms or side effects that are related to tRNS as follows: (1) absent; (2) mild; (3) moderate; and (4) severe.

### 2.6. Sample Size Considerations

The sample size was calculated by a priori analysis in G * Power, version 3.1.9.7 (The G*Power Team, Düsseldorf, Germany).

Because the design of this project has never been employed in children and adolescents with MLD, we could refer only to a pilot study that determined the effects of tRNS, combined with cognitive training, in children with MLD [66]. However, the study included 12 children with a medium effect size but differs in several aspects from ours: the number of tRNS sessions, number of groups, and cognitive training. To be conservative and start from these premises, we calculated the expected effect size (f) to low and estimated it at 0.15.

With an estimated f = 0.15, α value = 0.05 (i.e., probability of false positives of 5%), and β = 0.80 (i.e., at least 80% power), the sample size that was required for repeated-measures analysis of variance (RM ANOVA) with 3 groups (Frontal Group vs. Parietal Group vs. Sham Group) and 4 measurements (T0 vs. T1 vs. T2 vs. T3) was 78 (i.e., 26 per group). Considering a 30% dropout rate in the follow-ups, we will plan to recruit a total of 102 participants (i.e., 34 per group).

### 2.7. Safety Considerations

There are minimal risks that are associated with participation in the study. The potential risks are as follows:

(1) Transcranial random noise stimulation. Considering the paucity of safety data on tRNS, this technique is considered safe, as supported by a recent study in juvenile mice [61]. Previous studies [90,91] have shown that tRNS with the current intensity that we use here is less likely to be perceivable. Compared with tDCS, tRNS has the advantage of having higher cutaneous perception thresholds and lower response rates [90]. Adverse effects will be registered throughout the study. The experimenter will also follow participants for adverse effects after the end of the study.
(2) Cognitive training/assessment. There is a risk that participants will find the tasks to be challenging, fatiguing, or boring. Should this occur, participants can take a break at any time or can discontinue the testing. Research staff will explain what to do and how to perform the tasks during study visits.

### 2.8. Protection of Risks

To minimize any risk that is associated with tRNS, participants will be monitored throughout the stimulation sessions and asked to report any discomfort. If the scalp sensation is uncomfortable, the stimulation will be stopped. In the event of a headache, the stimulation will be stopped. All tRNS sessions will be administered and supervised continuously by a trained experimenter. There are no reports of seizures having been induced by tRNS in human participants. However, to avoid any chance of seizure, a prior history of neurological disorders is an exclusionary criterion for our study, and no participants will have a history of seizure. The risks that are associated with cognitive training and assessment are minimal. Nevertheless, breaks will be offered if participants experience frustration with the tasks.

### 2.9. Missed Sessions and Early Termination of Participation

Participants who, for any reason, miss a scheduled brain stimulation session or assessment will be given the opportunity to make it up the following day (including weekends if necessary). If they withdraw or are removed from the study at any phase, they will discontinue all research-related activities, including termination of the tRNS and all assessments that are directly related to the study. Clinical care will be unaffected.

### 2.10. Study Monitoring and Data Management

The principal investigator (or the ethics committee) will identify a study monitor who is assigned to follow this study per this Clinical Trial Protocol [European guidelines for Good Clinical Practice (CPMP/ICH/135/1995) and Decree-Law Italian Minister of Health, 15 July 1997]. The Investigator agrees to provide reliable data and all information that is requested by the Protocol in an accurate and legible manner according to the instructions that are provided and ensure direct access to source documents to ethics committee representatives. If any particular circuit must be defined, attention should be paid to the confidentiality of the participants’ data that are to be transferred.

The principal investigator may appoint other individuals as deemed appropriate as subinvestigators to assist in the conduct of the Clinical Trial per the Clinical Trial Protocol. All sub-investigators shall be timely appointed and listed. The subinvestigators will be supervised by and under the responsibility of the principal investigator. The principal investigator will provide them with a Clinical Trial Protocol and all necessary information.

The participants’ personal data will be anonymous and coded. The hard files will be placed in a closed drawer. The database will be protected by password. The investigators will allow the monitoring of the data at an appropriate frequency. The original documents will be available at any time to be verified by the clinical monitor and regulatory authority.

## 3. Data Analysis

### 3.1. EEG Preprocessing

EEG preprocessing will be performed separately for data that are collected before (T0) and after the interventions (T1, T2, T3), with the examiner being blinded to the stimulation conditions for all preprocessing steps. First, the EEG file will be imported into MATLAB (R2020b, The Mathworks) using the EEGlab toolbox (eeglab2021.0) [92], and channels will be located on the scalp model using the NE_EEGLAB_NIC_Plugin_v1.9 plugin (Neuroelectrics). Data will be band-pass-filtered from 1 Hz (high-pass) to 70 Hz (low-pass). The line noise (~50 Hz) will be removed. Next, the data will be divided into windows of 1 sec (epochs), and EEG epochs with muscle, electrode, and blinking artifacts will be manually removed. Independent component analysis will be conducted, and any period of nonindependence between components will be identified by eye. The spectral power for each epoch in four frequency bands [theta (θ) = 4–7.99 Hz; alfa (α) = 8–12.99 Hz; beta (β) = 13–29.99 Hz; gamma (γ) = 30–50 Hz] will be computed and averaged across epochs for each participant.

### 3.2. Statistical Analysis

The Shapiro–Wilk test will be used to test the normality of the data, and Levene’s test will be performed to analyze the homogeneity of the variances. When the data are normally distributed and the assumption of homogeneity is not violated, parametric analyses will be computed. When one assumption is not met, nonparametric tests will be conducted or log-transformation of the distribution will be applied, if appropriate. When appropriate, sphericity will be verified by Mauchly’s sphericity test. When sphericity is not met, Greenhouse–Geisser correction will be applied.

Chi-square analysis will be used to compare groups with regard to demographics, blindness, and safety measures (categorical variables).

Linear mixed-effects models, which account for within-subject correlations more optimally than analysis of variance and automatically handle missing values, allowing maximum use of available data [93], will be run to compare groups for primary (continuous variables: number line subtest) and secondary outcomes (continuous variables: arithmetic, neuropsychological, and psychological measures). The R package lme4 v1.1–17 [94,95], which estimates model parameters using restricted maximum likelihood estimation and computed *p*-values per Satterthwaite’s method [96], will be used. The linear mixed-effects models will include Group (Frontal, Parietal, and Sham) and Time (T0, T1, T2, T3) as fixed factors and Participants as the random factor. Baseline performance will be included as a covariate in our model, because it allows us to make better adjustments for minor differences in pretreatment means. The same analysis will be performed for the training accuracy data, including Group (Frontal, Parietal, and Sham) and Day (from 1 to 10) as fixed factors and participants as the random factor.

Pearson’s correlation will be used to determine whether and the extent to which arithmetic improvements are related to changes in neuropsychological measures, psychological measures, and spectral power of EEG frequency bands at T1, T2, and T3 (from T0).

The statistical results will be corrected for multiple comparisons by Bonferroni’s correction where appropriate.

### 3.3. Hypothesis and Expected Results

We expect to observe the following:

(1) The Parietal, Frontal, and Sham groups will not differ in blindness or safety measures;
(2) The Parietal and Frontal groups will experience significant improvements in the primary outcome (Number Line subtest accuracy) and arithmetic and neuropsychological measures at T1 compared with Sham group and that such improvements will persist at T3. The Parietal and Frontal groups will also significantly improve their training accuracy across the 10 days versus the Sham group. We do not have a directional hypothesis on the differences between the 2 active groups (Parietal and Frontal);
(3) The Parietal and Frontal groups will significantly improve their psychological measures at least at T3 compared with the Sham group;
(4) In the Parietal and Frontal Groups, arithmetic changes will correlate strongly and positively with changes in neuropsychological and spectral power EEG measures at least at T1 and with changes in psychological measures at least at T3.

## 4. Discussion

We have described the rationale and design of a trial that aims to determine whether tRNS, combined with cognitive training, will have a clinically meaningful impact on math and math-related (e.g., working memory, mental rotation) abilities and psychological and neurophysiological measures in children and adolescents with MLD. This study will constitute the first attempt to prove the safety and feasibility of multiple (e.g., 10 sessions) and consecutive sessions of tRNS in the pediatric population. In addition, the project will determine the most effective placement of tRNS electrodes (Frontal vs. Parietal) compared with placebo (Sham) in terms of behavioral and neurophysiological long-term improvement. The results will represent a significant step toward clinical translation in the field.

A unique facet of this study relies on the potential synergy of multitarget interventions, with one each serving as “endogenous” activation, targeting behavior through cognitive training, and “exogenous” neuromodulation [55] through its direct neurophysiological effect on brain regions that surround the electrodes on distal cortical areas. Accordingly, tRNS would act to desynchronize pathological cortical rhythms via enhancing the neural signals detection and thus improving the neural processing and the related behavior [58,60,97]. In our case, tRNS has the potential to prompt math training-induced neuroplasticity, facilitating brain activity in the frontal or parietal network during cognitive training.

An additional unique feature is that our study will involve repeated consecutive sessions of tRNS combined with cognitive training as multiple sessions of transcranial electrical stimulation gave higher chances of cumulative biological effects over time [98]. The decision of a multi-sessions protocol was made after cautiously considering neurophysiological and preliminary data that supported evidence for tolerability and safety of this technique in children at this stage [66,99,100]. In line with our considerations, a recent study [73] applied five-days of tRNS combined with cognitive training in children with attention deficit hyperactivity disorder and the authors revealed no clinical-meaningful side effects or related safety-issues at any follow-up.

Although tDCS is the most widely used neuromodulatory technique during developmental ages [101,102], in the last few years tRNS popularity has sharply increased. While tRNS appears to have at least the same potential long-term effects as anodal tDCS [62], it is easier to blind than tDCS, making it preferable for double-blind studies [90,100]. Of importance, a number of studies showed that tRNS would induce more pronounced and consistent enhancements compared to tDCS in perceptual [91] and cognitive domains [103], as well as in clinical populations [73].

The selection of tRNS parameters was based (1) on a single study using tRNS in children with MLD [66] and (2) on evidence of the beneficial and safe effects of high-frequency tRNS in the motor [104,105], sensory-perceptual [60,106,107], and cognitive [108,109,110] domains and, specifically, in arithmetic tasks [62,65,66]. Regarding the current intensity, 1 mA is well tolerated in adults, without adverse effects [62,63,64,65,72], but guidelines for children recommend applying at least half of that for adults [111]. The decision to apply 75% of 1 mA was made after considering the parameters that influence the current distribution and density at the site of stimulation, such as a thinner scalp, less cerebrospinal fluid, and the smaller head size of the pediatric population [111,112,113].

Regarding the frequency band, tRNS can encompass a full-frequency range (typically from 0.1–640 Hz) or can be delivered at low or high frequency (by convention, respectively, from 0.1–100 Hz and 101–640 Hz) [58]. In our study, we decided to apply a high-frequency band of stimulation, given that only higher frequencies (100–500 Hz) of tRNS generate consistent excitability that lasts for up to 60 min after stimulation [58].

Its multilevel assessment is also a unique feature of this randomized clinical trial. Measuring the behavioral, psychological, and neurophysiological aspects across participants is particularly relevant in studying the effectiveness of tRNS. The multiaspect measurements will allow us to detect the direct effects of the interventions on the main targeted areas and, importantly, the translational benefits for the psychological function of children. Considering that its neurophysiological mechanisms of action are unknown [56], the recording of EEG data before and after the interventions will be used as a proxy of neuroplasticity and as a reliable neurophysiological marker for treatment responders. Evaluating the impact of this intervention to the psychological well-being of participants is not trivial as it could accelerate its translation to the clinical setting.

## 5. Conclusions

Detailed reporting of clinical trial protocols ensures the vigor, soundness, and reproducibility of research. We firmly believe that this clinical trial will produce reliable and positive results to accelerate the validation of brain-based treatments for MLD, with a potential impact on the quality of life of such patients. Caring for atypically developing brains through brain-directed interventions could shift the developmental trajectories of mental and cognitive functions in a supportive manner.
